# Percutaneous Emergence of *Gnathostoma spinigerum* Following Praziquantel Treatment

**DOI:** 10.3390/tropicalmed4040145

**Published:** 2019-12-14

**Authors:** Sarah G. H. Sapp, Monica Kaminski, Marie Abdallah, Henry S. Bishop, Mark Fox, MacKevin Ndubuisi, Richard S. Bradbury

**Affiliations:** 1 Parasitic Diseases Branch, Division of Parasitic Diseases and Malaria, Center for Global Health, Centers for Disease Control and Prevention, Atlanta, GA 30029, USA; hsb2@cdc.gov (H.S.B.); nyg3@cdc.gov (M.F.); nrm6@cdc.gov (M.N.); rbradbur76@gmail.com (R.S.B.); 2 New York City Health and Hospitals Corporation, New York, NY 10013, USA; Monica.Kaminski@downstate.edu (M.K.); marie.abdallah@nychhc.org (M.A.); 3 Oak Ridge Institute for Science and Education, Oak Ridge Associated Universities, Oak Ridge, TN 37830, USA

**Keywords:** gnathostomiasis, schistosomiasis, imported helminthiasis, praziquantel

## Abstract

A Bangladeshi patient with prior travel to Saudi Arabia was hospitalized in the United States for a presumptive liver abscess. Praziquantel was administered following a positive *Schistosoma* antibody test. Ten days later, a subadult worm migrated to the skin surface and was identified morphologically as *Gnathostoma spinigerum*. This case highlights the challenges of gnathostomiasis diagnosis, raising questions on potential serologic cross-reactivity and the possible role of praziquantel in stimulating outward migration of *Gnathostoma* larvae/subadults.

## 1. Introduction

Gnathostomiasis is a foodborne zoonosis with diverse and sometimes serious clinical outcomes. Transmission occurs via consumption of advanced third-stage (AL3) larvae encysted in undercooked meat of intermediate or paratenic hosts, commonly freshwater fish, frogs, snakes, and fowl. Larvae undergo an invasive course of migration in the aberrant human host after penetrating the gastrointestinal wall, first migrating to the liver parenchyma and then other organs [1]. The usual presentation is cutaneous gnathostomiasis, frequently preceded by a nonspecific prodrome due to larval migration. Other presentations include urogenital, visceral, ocular, and neurological gnathostomiasis [1,2]. Management can be challenging with frequent relapses or treatment failures [3]. 

The known zoonotic species are *Gnathostoma spinigerum, G. hispidum, G. doloresi, G. nipponicum, G. binucleatum,* and possibly *G. malaysiae* [1,2]. Among these, *G. spinigerum* is the best-studied with the broadest occurrence; this species is endemic across Southeast Asia, East Asia, India, Africa, and possibly Australia [1]. Gnathostomiasis has been recognized as a parasitic infection of travelers and refugees from endemic regions [2,4], however, awareness among general practitioners in nonendemic countries may be limited.

## 2. Case Report 

In June 2018, a 46-year-old female originally from Bangladesh (emigrated to the USA in 2017) presented to her primary care physician with complaints of diarrhea. The patient had traveled to Saudi Arabia for ten days and developed symptoms one week after returning to the USA. She was prescribed a 7-day course of metronidazole, though she only complied for 2 days due to religious fasting. Two weeks following symptom onset, the patient was admitted to the hospital with diffuse intermittent abdominal pain that began two days earlier and continuing diarrhea. She reported epigastric and right upper abdominal quadrant (RUQ) pain and increased belching. No other signs/symptoms were reported or observed.

Upon admission she was afebrile and had stable vital signs. Physical examination revealed mild discomfort to deep palpation of the epigastrium and RUQ. Her white blood cell (WBC) count was elevated at 20.64 × 10^9^/L (normal (4.70–10.30) × 10^9^/L) with 57.8% eosinophils, 18.8% neutrophils, 19.7% lymphocytes, and 0.5% basophils. A CT scan of the abdomen showed diffuse gastric wall thickening with mild adjacent inflammatory change, suggestive of gastritis, and a hypodensity in the left lower liver measuring 2.2 cm with a rim enhancing wall, suspicious for abscess. Metronidazole and ceftriaxone treatment was initiated. Interventional radiology was consulted for liver abscess drainage, but the procedure was deferred in view of the small size. The abdominal pain resolved on the second day of hospitalization, but the patient remained admitted for continuation of IV antibiotic treatment.

On the third day of hospitalization, serum and stool specimens were collected and sent for testing for various parasitic etiologies, including schistosomiasis, to determine the cause of peripheral eosinophilia and liver abscess. Antibody tests for *Entamoeba histolytica*, *Strongyloides,* and *Toxocara* and a pan-filarial assay were negative. Three of four stool examinations were negative (one positive for *Blastocystis*). The patient was discharged on day 8 following placement of a peripherally-inserted central catheter for the continuation of intravenous ceftriaxone (6-week course) and oral metronidazole for liver abscess treatment. 

**Figure 1:**
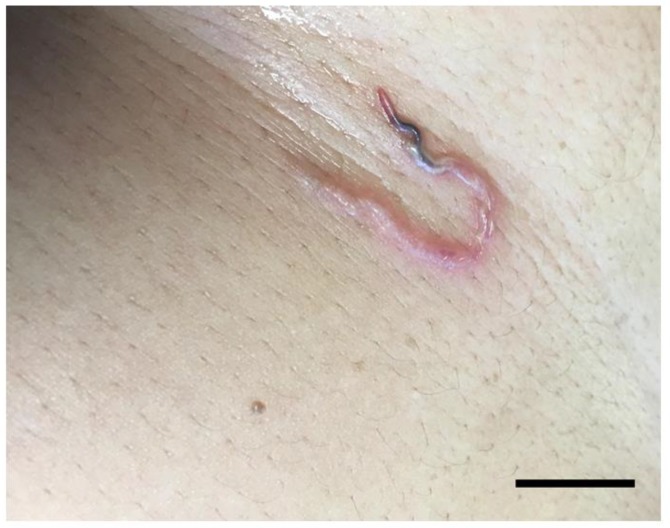
Serpiginous track showing the emerging subcutaneous *Gnathostoma spinigerum*. (Bar = approximately 1 cm).

Five weeks following discharge, a RUQ sonogram showed abscess resolution, however, blood work revealed persistent WBC elevation (17.66 × 10^9^/L) with 46.1% eosinophils, 29.0% neutrophils, 20.6% lymphocytes, 2.9% monocytes, and 0.5% basophils. She was advised to continue ceftriaxone/metronidazole for another week. A few days later, a low positive result was returned for the previously-ordered *Schistosoma* antibody test (FAST-ELISA value 13.0 (0–10 normal)); ceftriaxone/metronidazole was ceased and praziquantel (40 mg/kg in 2 doses taken in one day) was prescribed. Ten days after praziquantel treatment, the patient reported epigastric pain with localized rash, pruritus, and hyperesthesia. Clinical examination identified a serpiginous track with an emerging worm over the upper abdomen (Figure 1) which was extracted. Photos of the parasite were submitted for telediagnosis to the Centers for Disease Control and Prevention (Atlanta, Georgia, USA). The specimen was stored in 70% ethanol and shipped for morphologic examination. 

**Figure 2:**
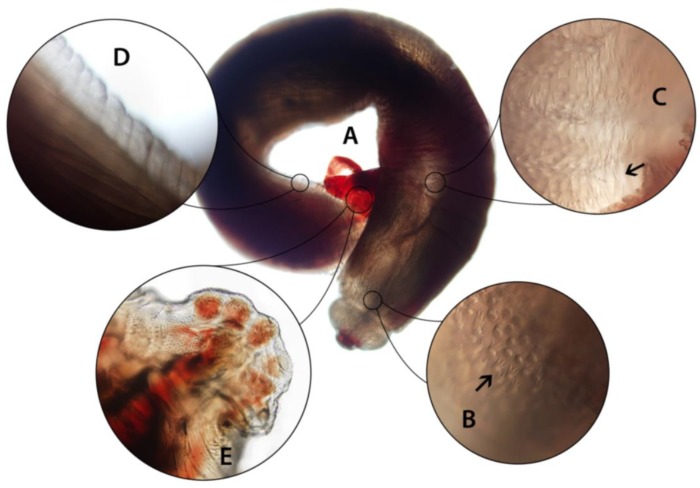
Subadult male *Gnathostoma spinigerum* extracted from the patient. (**A**) Whole worm, measuring 0.66 cm; (**B**) short, three-pointed spines just behind cephalic bulb; (**C**) longer spines on anterior half of body; (**D**) aspinous area of the posterior body; (**E**) caudal alae showing round pedunculate papillae and surface texture with simple spines. Photos of spines taken under 200× magnification.

By microscopy, the gross appearance and the presence of a cephalic bulb and broad caudal alae (Figure 2) were characteristic of a subadult male *Gnathostoma*. The total length was 0.66 cm; other morphometric characteristics are summarized in Table 1. The cephalic bulb had two lips and cephalic spines were simple, arranged in eight alternating rows. The blunt posterior was difficult to assess in detail due to the position in which the worm was fixed; caudal alae were broad and rounded. The extremities were deep red in color, and body spines began after the cephalic bulb. Anterior spines were mostly tricuspidate, occasionally quadricuspidate, with points of roughly even length. Further posteriad, spines became elongate and bicuspidate and eventually became single points before an aspinous area at ~60% of the distance from the anterior. Sparse, simple spines were present on the interior portion of caudal alae (Figure 2).

Based on detailed microscopic examination and characteristics of the body spines, this worm was identified as *Gnathostoma spinigerum.* The shape and distribution of spines were sufficient to rule out other zoonotic *Gnathostoma* spp. using published descriptions. PCR was also attempted on a small fragment of the worm, but insufficient DNA was extracted and amplification was unsuccessful. The patient was treated with ivermectin (0.2 mg/kg, 2 days) following confirmation of the diagnosis, resulting in resolution of symptoms and eosinophilia. A follow-up abdominal CT scan one month later was normal. 

## 3. Discussion

We identified an imported case of cutaneous gnathostomiasis caused by a subadult male *G. spinigerum* with some interesting characteristics. *Gnathostoma* spp. diagnosed in cases of deeper tissue involvement (e.g. brain, urogenital, liver) are typically of a larval stage, but worms from cutaneous cases may show a variable degree of maturation, although never reaching sexual maturity [2,5]. Recovery of the intact, subadult worm allowed for species determination based on body spines, which is more straightforward than on advanced third-stage larvae (AL3). For example, all but one zoonotic *Gnathostoma* species have AL3 with four rows of cephalic hooklets, and body spines are not sufficiently developed [1,5]. Histological sectioning allows examination of intestinal cell morphology, but this may be difficult to distinguish except in ideal lateral sections [6]. 

Spontaneous percutaneous emergence is relatively rare in gnathostomiasis cases; however, this may apparently be induced by drug treatment. Albendazole was demonstrated to stimulate outward migration of *G. spinigerum* larvae in a clinical trial involving Thai patients presenting with serologically-confirmed cutaneous gnathostomiasis [7]. Emergence did not occur among 40 patients in the placebo-treated group but was recorded three times in the group receiving albendazole (400 mg, 2× daily for 12 days). These events occurred 8–14 days following treatment and were preceded by erythematous linear or papular lesions on areas where worms eventually emerged. In two patients, the larvae were superficial enough that they were removed by squeezing or with a needle [7]. Percutaneous emergence has also been noted sporadically following single-dose ivermectin treatment (0.2 mg/kg), though more detailed clinical information is unavailable [8,9]. The development of migratory, swollen nodules was also reported in association with interferon alpha-2b injections for hepatitis C, although in this patient the worm did not emerge percutaneously until after nine months of therapy [10]. While additional targeted studies will be necessary to definitively implicate praziquantel as a larval migratory stimulus, the emergence of the worm in this patient mirrors the presentation and timing observed in the albendazole trial, suggesting that the emergence could possibly have been stimulated by the drug. The mechanism by which outward migration is stimulated is not known, including whether it represents directional or random motility; it is also not clear why a drug with no appreciable nematocidal activity would induce emergence. The nematode cuticle is believed to confer resistance to the calcium-channel-disrupting effects observed in Platyhelminthes, but nematodes may still uptake small amounts of praziquantel orally [11,12]. 

As some cases of gnathostomiasis have also been initially misdiagnosed as schistosomiasis [13,14], it is important that clinicians consider the likelihood of exposure, clinical features, and serologic results in differential diagnosis. However, the overlapping geographic ranges of these parasites, nonspecific clinical manifestations, and difficulty of serologic interpretation present a challenge—particularly if patients report complex travel history. Our patient presented with eosinophilia and was positive for *Schistosoma* antibody, prompting praziquantel treatment. She had recently returned from Saudi Arabia, but the testing was within the five-week minimum period required for seroconversion in schistosomiasis [15,16]. Exposures within a year or two prior were unlikely since Bangladesh is schistosomiasis nonendemic [17]. This raises the possibility that the *G. spinigerum* infection generated antibodies that reacted with the schistosomiasis antigen in the FAST-ELISA. Some schistosomiasis seroassays have known cross-reactivity with nematode infections, and anti-*Gnathostoma* antibodies were shown to cross-react in the *Schistosoma mekongi*-specific *Smk*AWA ELISA [15]. Similarly to our case, serologic testing for *Schistosoma mansoni* in one reported gnathostomiasis case from Brazil was weakly positive, which lead to an initial incorrect diagnosis of schistosomiasis [14]. The low value of the FAST-ELISA result, lack of follow-up, and negative stool examination suggest that cross-reactivity was likely in our patient’s case. Alternately, the low level of *Schistosoma* antibody could possibly indicate a very old infection; it unknown if the patient had visited Saudi Arabia on multiple occasions prior to the reported visit. Overall, these findings highlight the importance of investigating potential serological cross-reactivity created by *Gnathostoma* infections, which may go undetected and mislead treatment decisions. Adding to the diagnostic challenge presented by gnathostomiasis is the very limited availability of serologic testing for *Gnathostoma* spp. (not available in the United States). As far as clinical evidence, gastric wall and liver involvement observed in this patient are more consistent with gnathostomiasis, as migration of *Gnathostoma* larvae involves the stomach penetration and liver parenchyma invasion and long latent periods (months to years) can occur, supporting the notion that the *G. spinigerum* was acquired some time ago in the endemic region [1,3,6]. 

Further research is necessary to understand the relationship between *Gnathostoma* emergence and praziquantel. Drug-induced stimulation of worm migration presents a hypothetical risk if such stimulation represents an increase in random migration. Larvae could perhaps invade deeper tissues rather than emerging through skin, with more dire clinical outcomes. Clinicians should be aware of the possibility of “uncovering” latent *Gnathostoma* infections following anthelmintic treatment in patients from endemic regions. 

## Figures and Tables

**Table 1:** Morphometric characteristics of the subadult *Gnathostoma spinigerum* male extracted from the patient.

Aspect	Size
Total length	0.66 cm
Cephalic bulb maximal width	575 µm
Cephalic bulb length	300 µm
Width immediately behind cephalic bulb	675 µm
Width at midbody	950 µm
Width before posterior	700 µm
Caudal alae	260 µm widest 210 µm narrowest
